# Integrated analysis of intratumoral biomarker and tumor-associated macrophage to improve the prognosis prediction in cancer patients

**DOI:** 10.1186/s12885-023-11027-6

**Published:** 2023-06-27

**Authors:** Ming-Da Wang, Hao Xiang, Tian-Yu Hong, Abudurexiti Mierxiati, Fei-Hu Yan, Ling Zhang, Chao Wang

**Affiliations:** 1 Shanghai Health Commission Key Lab of Artificial Intelligence-Based Management of Inflammation and Chronic Diseases, Gongli Hospital, Navy Medical University, 219 Miaopu Road, Shanghai, 200135 China; 2 Department of Hepatobiliary Surgery, Eastern Hepatobiliary Surgery Hospital, Navy Medical University, Shanghai, 200433 China; 3 The Third Affiliated Hospital of Zunyi Medical University, The First People’s Hospital of Zunyi), Guizhou, 563000 China; 4 Department of Colorectal Surgery, Shanghai Changhai Hospital, Navy Medical University, Shanghai, 200433 China; 5 Department of Obstetrics and Gynecology, School of Medicine, Chengdu Women’s and Children’s Central Hospital, University of Electronic Science and Technology of China, Sichuan, 610000 China; 6 Department of Urinary Surgery, Gongli Hospital, Navy Medical University, Shanghai, 200135 China

**Keywords:** Hepatocellular carcinoma, Tumor-associated Macrophage, HBx, CD68, Prognosis, Predictive accuracy

## Abstract

**Background:**

The lack of effective and accurate predictive indicators remains a major bottleneck for the improvement of the prognosis of patients with hepatitis B virus (HBV)-related hepatocellular carcinoma (HCC). Hepatitis B virus X (HBx) has been widely suggested as a critical pathogenic protein for HBV-driven liver carcinogenesis, while tumor-associated macrophage (TAM) infiltration is also closely related to the tumorigenesis and progression of HCC. However, few studies have determined whether combining HBx expression with TAM populations could increase the accuracy of prognostic prediction for HBV-related HCC.

**Methods:**

The study cohort enrolling 251 patients with HBV-related HCC was randomly split into a training and a validation group (ratio 1:1). The expression levels of HBx and TAM marker CD68 in HCC samples were detected by immunohistochemistry. Kaplan–Meier curves, Cox regression and Harrell’s concordance index (C-index) analysis were conducted to evaluate the prognostic significance of these indicators alone or in combination.

**Results:**

The expression level of HBx was strongly correlated with CD68^+^ TAM infiltration in HCC tissues. Elevated HBx or CD68 expression indicated poorer overall survival (OS) and progression-free survival (PFS) after hepatectomy, and both of them were independent risk factors for postoperative survival. Meanwhile, patients with both high HBx and CD68 levels had worst clinical outcomes. Moreover, integrating HBx and CD68 expression with clinical indicators (tumor size and micro-vascular invasion) showed the best prognostic potential with highest C-index value for survival predictivity, and this proposed model also performed better than several conventional classifications of HCC.

**Conclusion:**

Combining the expression of intratumoral HBx, CD68^+^ TAM population and clinical variables could enable better prognostication for HBV-related HCC after hepatectomy, thus providing novel insights into developing more effective clinical prediction model based on both molecular phenotypes and tumor-immune microenvironment.

**Supplementary Information:**

The online version contains supplementary material available at 10.1186/s12885-023-11027-6.

## Introduction

Hepatocellular carcinoma (HCC) ranks as the sixth most frequent malignant tumors and the third leading cause of death from cancer worldwide, making it a major public health challenge with enormous economic burdens [1]. Hepatitis B virus (HBV) infection is the predominant etiological factor of HCC particularly in Southeast Asia and sub-Saharan Africa, where HBV-related HCC is prevalent [2]. More than half of the world’s total HCC cases occur in China, with an estimate of 420,000 newly diagnosed HCC patients and 390,000 deaths in 2018 [3]. Although surgical resection is the mainstay treatment modality for HCC and have the potential to cure patients at earlier stages, the overall prognosis of HCC patients is still far from satisfaction, with a high recurrence rate of up to 70% within 5 years after surgery [1, 4]. Difficulties in early diagnosis and prognostic prediction for HCC, as well as the limited knowledge of the great heterogeneity and molecular mechanisms underlying HCC carcinogenesis largely impeded the improvement of long-term prognosis for HCC patients. Thus, to improve surgical results and to prolong postoperative survival of these patients, it is imperative to identify more reliable and promising indicators or biomarkers for prognostication and monitoring HCC recurrence after surgery.

It has been reported that multiple viral factors are involved in the development and progression of HBV-related HCC. Among them, hepatitis B virus X (HBx) protein, which is encoded by a 1.1 kb fragment of the HBV genome, has been implicated in viral replication and viral-mediated liver carcinogenesis [5, 6]. It is also a key transactivator that regulates various cellular genes involved in tumorigenesis [7–9]. Previous studies including ours have revealed that aberrant overexpression of HBx was significantly associated with aggressive clinicopathological characteristics and poor prognosis of patients with HBV-related HCC [10–13], making it a potential prognostic indicator and therapeutic target for HCC management. However, the tremendous heterogeneity of HCC makes it difficult for a single HBx oncogene to comprehensively reflect the malignant behaviors of HCC and accurately predict clinical outcomes. This has prompted us to further explore whether combining HBx with additional molecules or biomarkers could better predict prognosis and guide treatment for HBV-driven HCC patients.

The tumor immune microenvironment, which consists of a wide variety of immune cell subtypes and non-cellular elements, has gained extensive attention for its vital effects on tumorigenesis and development [14, 15]. As an indispensable part of the microenvironment, tumor-associated macrophages (TAMs) have been shown to play a critical role in the occurrence and progression of malignant tumors, including HCC [16–18]. It was suggested that specific TAM subtypes can be recognized as prognostic indicators for tumors, and multiple molecular biomarkers have been used for the identification of TAM populations [19, 20]. Among them, CD68 is one of the well-accepted TAM-specific biomarkers, and the association of CD68^+^ TAMs infiltration with poor clinical outcomes has been well documented both in HCC and other malignancies [21–26]. As for HBV-mediated carcinogenesis, although HBx plays a critical role in a range of physiological and pathological processes, very few studies have reported the interplay between HBx expression and the tumor immune microenvironment or the regulation of HBx to the infiltration of TAM subsets in the context of HCC. Also, whether combing the expression level of HBx and TAM biomarker CD68 could better stratify postoperative prognosis in patients with HBV-related HCC remains obscure.

Thus, this study sought to determine the relationship between high expression of HBx and CD68^+^ TAMs infiltration in HBV-related HCC tumor tissues, and further evaluated the prognostic value of integrating HBx and CD68 for survival prediction in a cohort of HCC patients with chronic HBV infection. Meanwhile, the clinical significance of combining the HBx-CD68-based prognostic model with currently used clinical indicators in predicting postoperative prognosis of patients with HBV-related HCC was also investigated. Our data may provide new insight into understanding of the cross-talk between HBx expression and TAMs infiltration in HCC progression, and also identify a promising prognostic index for HBV-related HCC patients after liver resection.

## Materials and methods

### Patients and samples

Data on 251 consecutive patients who underwent curative-intent liver resection for HBV-related HCC at Eastern Hepatobiliary Surgery Hospital between March 2013 and September 2016 were retrospectively collected and analyzed. The diagnosis of HCC for all surgical specimens was confirmed by histopathological examinations after surgery. Curative resection was considered as complete removal of all visible lesions with microscopic negative margins (R0 resection). All patients were positive for serum HBV surface antigen (HbsAg), and chronic HBV infection was defined as HbsAg positivity for more than 6 months. Patients who (a) had portal vein tumor thrombus; (b) had received any anti-tumor treatment prior to surgery or (c) had other etiologies of HCC except for HBV infection were excluded. All enrolled patients were randomly classified into the training (*n* = 126) and validation (*n* = 125) sets at a ratio of 1:1. This study was carried out adhering to the guideline of the Reporting Recommendations for Tumor Marker Prognostic Studies (REMARK) [27]. Approval was granted by the Ethics Committee and Institutional Review Board of the hospital. Written informed consent was obtained from all participants for the use of tissue samples and clinical data for research purpose. Patients’ demographic and clinical data including age, gender, positivity of HBV envelope antigen (HbeAg), presence of liver cirrhosis, Child–Pugh grading, Tumor Node Metastasis (TNM) stage, preoperative serum α-fetoprotein (AFP) level, largest tumor size, tumor number, tumor satellites, tumor differentiation and microvascular invasion (MVI) of both cohorts are summarized in Supplementary Table 1. The predictive endpoints were overall survival (OS) and progression-free survival (PFS). OS was calculated from the date of surgery to patient’s death or final follow-up, while PFS was defined as the time from surgery to first documented recurrence or disease progression. Routine laboratory and imaging examinations including liver function, serum AFP level and abdominal ultrasound were regularly scheduled in all patients who underwent hepatectomy for HCC every 2 to 3 months after discharge from hospital. Detailed follow-up program, diagnosis of postoperative HCC recurrence and the relevant treatment modalities have been clearly stated in our previous study [4].

### Immunohistochemistry (IHC)

HCC tissue microarray (TMA) blocks were constructed by using paraffin-embedded tissue samples from 251 patients evaluated, and then sliced into 4 µm-thick sections for IHC staining as previously reported [11]. Briefly, the TMA slides were subjected to deparaffinication, rehydration and then processed for antigen retrieval. Then the endogenous peroxidases and non-specific binding sites were blocked by incubating the slides with peroxidase blockers and normal goat serum, respectively, and the TMA sections were subsequently incubated with primary antibodies against HBx (1:200 dilution; rabbit polyclonal, ab39716, Abcam, Cambridge, UK) and CD68 (1:200 dilution; mouse monoclonal, ab53444, Abcam) at 4 °C in an incubator overnight. Then the slides were incubated with corresponding secondary antibodies and counterstained with hematoxylin after developing with diaminobenzidin. All slides were visualized and photographed under a light microscope (Olympus, Tokyo Japan) and TMA images were quantified double-blindly by two independent experienced pathologists. The staining index of HBx was determined by H-score, which was reported as an aggregate of the percentage of tumor cells staining positive multiplied by the staining intensity of the positive cells in given areas [28]. Based on this semi-quantitative grading method, staining intensity was ranged from 0 to 3, with 0 representing the negative or no detectable staining, score 1 weakly intense staining, score 2 moderate intense staining, and score 3 strongly intense staining. Similarly, the proportion of areas covered by positive staining cells was ranged from 0 to 100. As for CD68^+^ cells, the staining intensity was determined by evaluation of cell density, which was expressed as number of stained cells per mm^2^ (cell/mm^2^).

### Statistical analysis

All statistical calculations were conducted using SPSS Statistic version 21.0 (SPSS Inc., Chicago, IL, USA) and R-project software version 3.5.3 (http://www.r-project.org/). Continuous data were presented as mean ± standard deviation and compared using the two-tailed Student’s t test or Wilcoxon test, while discontinuous variables were displayed as number (proportion) and compared using the χ^2^ test or Fisher’s exact test. The OS and PFS curves were plotted using Kaplan–Meier method and compared using the log-rank test. Independent risk factors were identified using Cox proportional hazard regression models, and only those variables with a *P* value < 0.1 in the univariate analysis were enrolled into the multivariate analyses to predict postoperative survivals. Nomogram models were created using “rms” package of R software, based on the results of multivariate analysis. The cutoff values of HBx and CD68 were determined using time-dependent receiver operating characteristic (ROC) analysis with “survival ROC” package. The accuracy in predicting 5-year survival was assessed by the area under the curve (AUC) of ROC analysis. The discriminatory ability of any model or clinical indicators for survival prediction were calculated using Harrell’s concordance index (C-index) and calibration curves. A *P* value < 0.05 was considered statistically significant.

## Results

### Elevated HBx expression was closely linked to high density of infiltrating CD68^+^ TAMs in HBV-related HCC

**Fig. 1:**
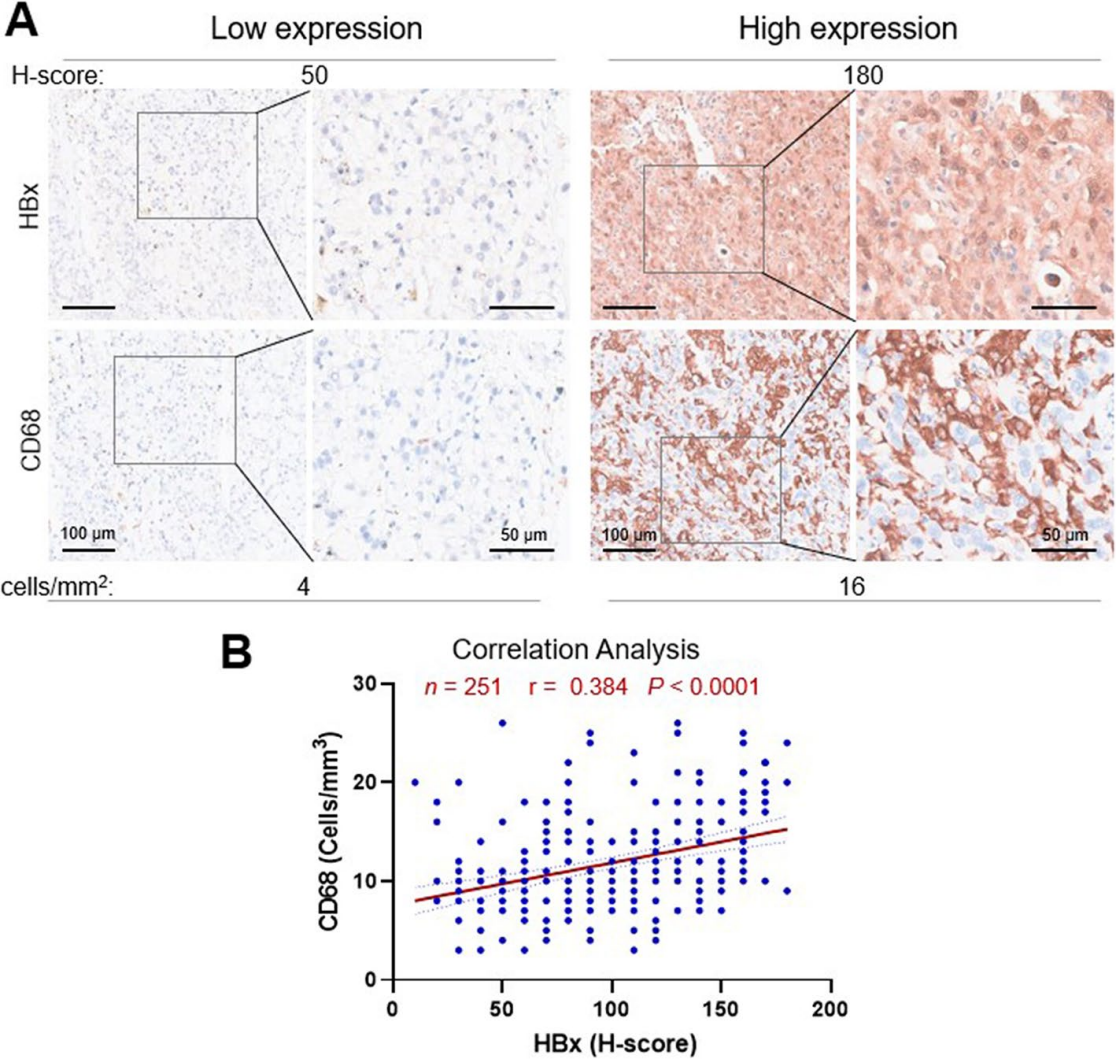
High expression level of intratumoral HBx was positively correlated with CD68^+^ TAM infiltration in HCC specimens. **A** Representative images of H&E and immunohistochemical staining of HBx and CD68 expression in HBV-related HCC samples. Scale bars: 100 (left) and 50 μm (right). **B** Pearson’s correlation analysis for the expression levels of HBx (H-score) and CD68 (cell density) in 251 HCC specimens

Considering the critical role of HBx in HBV-driven tumorigenicity and the emerging research focus of tumor immune microenvironment in HCC, it is worthwhile to illustrate the expression pattern and potential link between HBx and immune-related indicators, especially TAM surface markers, as well as their synthetic effect in the development of HBV-related HCC. Our previous study has analyzed the expression level of HBx mRNA in human HCC and paired adjacent non-tumoral tissues, with the results demonstrating that HBx was more frequently expressed in HCC tissues than in the peritumoral tissues [11]. To further validate these above results, an overall cohort comprising a total of 251 individuals with HBV-related HCC were randomly classified into the training and testing sets in a ratio of 1:1, and summary of the patients’ baseline characteristics of the two sets are shown in Supplementary Table 1. Then, IHC staining of the TMA sections from HCC patients were performed to detect the expression of HBx and CD68 in HBV-related HCC tissues (Fig. 1A). Although the expression levels of HBx and CD68 varied widely among all tumor samples, HBx expression was demonstrated to be strongly correlated with CD68 in human HBV-related HCC tissues, with a Pearson’s coefficient of 0.38 (*P* < 0.0001; Fig. 1B). These data provided evidence about the strong correlation between intratumoral HBx expression and CD68^+^ TAM infiltration during HBV-mediated liver carcinogenesis.

### Elevated expression of HBx or CD68 was predictive of poor postoperative prognosis of HBV-related HCC patients

**Fig. 2:**
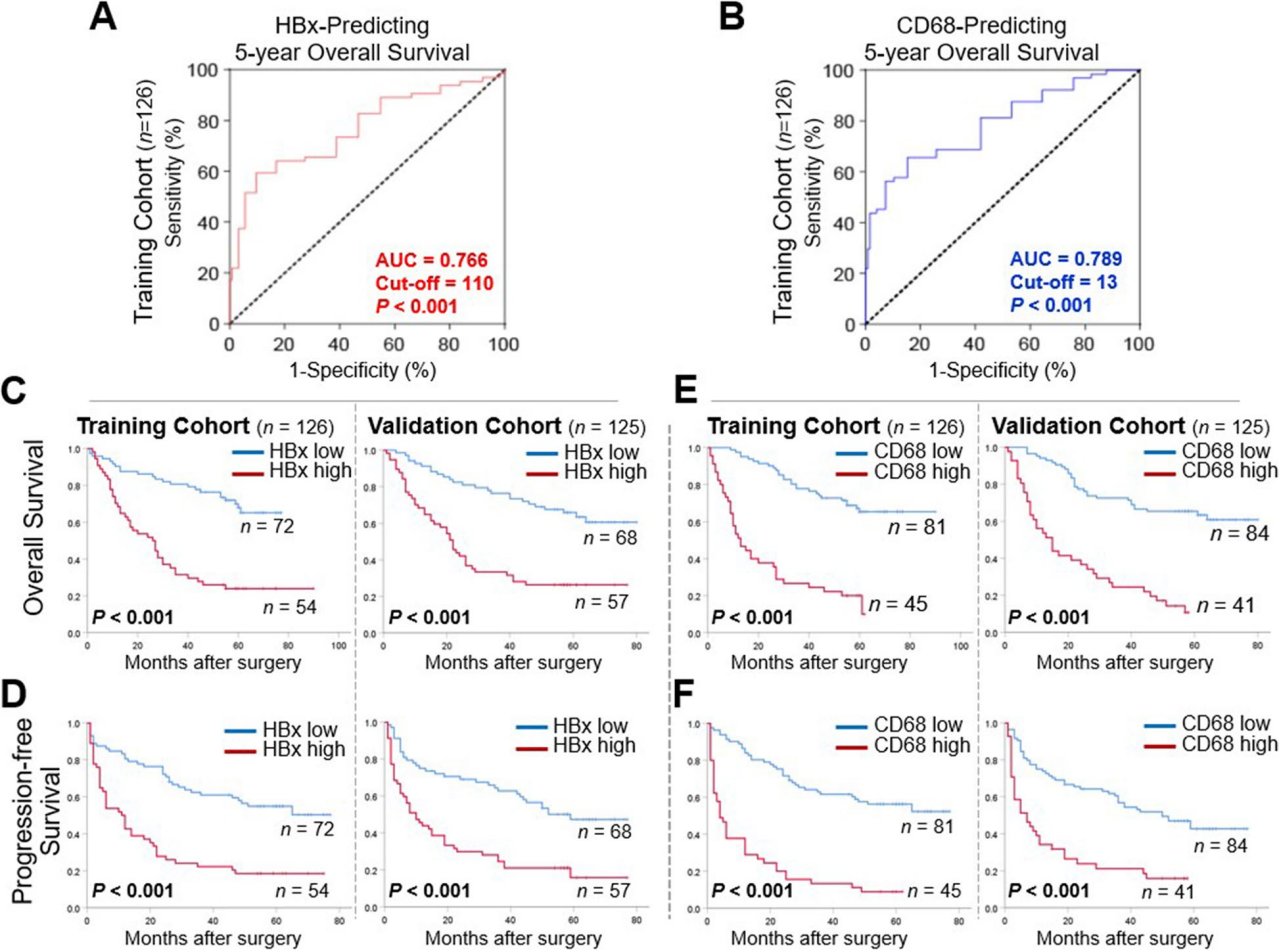
Either high HBx or CD68 expression was predictive of unfavorable long-term clinical outcomes after hepatectomy for HBV-related HCC. **A** ROC analysis of the optimal cut-off value of HBx to predict 5-year OS in training cohort (*n* = 126; cut-off H-score of HBx: 110, AUC = 0.766, *P* < 0.001). **B** ROC analysis of the optimal cut-off value of HBx to predict 5-year OS in training cohort (*n* = 126; cut-off cell density of CD68^+^ TAM: 13, AUC = 0.789, *P* < 0.001). **C**, **D** Kaplan–Meier curves of OS and PFS for HBV-related HCC patients according to the expression levels of HBx (high v.s. low) in training (*n* = 126) and validation cohort (*n* = 125). **E**, **F** Kaplan–Meier curves of OS and PFS for HBV-related HCC patients according to the cell density of CD68^+^ TAM (high v.s. low) in training (*n* = 126) and validation cohort (*n* = 125). ROC, receiver operating characteristic; TAM, tumor-associated macrophage; OS, overall survival; PFS, progression-free survival

Given the observed correlation between HBx and CD68 at the expression level in HCC samples, the functional roles of these two biomarkers, either alone or in combination, in the evaluation and prognostication of patients with HBV-related HCC after resection were further explored. Using 5-year OS as the major endpoint, the best cut-off value of the HBx and CD68 IHC score for survival prediction was determined by ROC analysis. As shown in Fig. 2A and B, the optimal cut-off values of HBx for predicting 5-year OS of patients in the training cohort was 110 (H-score; AUC = 0.766), while H-score of 13 was the best cut-off point for CD68 with an AUC of 0.789. Then, Kaplan–Meier survival curves were plotted to illustrate the prognostic value of HBx or CD68 in HBV-related HCC. Patients with either high HBx or CD68 expression showed significantly inferior OS and PFS compared to their counterparts. Similar findings were also observed in the validation cohort by using the same cut-off values established in the training cohort (Fig. 2C-F). Furthermore, univariate and multivariate Cox regression analyses were used to assess the independent prognostic factors associating with the clinical outcomes. As shown in Table 1, both HBx and CD68, as well as MVI and tumor size, were independently associated with worse OS and PFS of patients with HBV-related HCC in the training cohort. In addition, consistent results were observed in the validation and overall cohorts (Supplementary Table 2), confirming the prognostic value of either HBx or CD68 in evaluating post-surgical prognosis of patients with HBV-related HCC following hepatectomy.

**Table 1:** Univariate and multivariate Cox regression analysis of HBx, CD68 expression and clinical variables with overall survival and progression-free survival in training cohort (*n* = 126)

Characteristics	Overall survival				Progression-free survival			
	**Univariate**		**Multivariate**		**Univariate**		**Multivariate**	
	**HR (95% CI)**	***P* Value**	**HR (95% CI)**	***P* Value**	**HR (95% CI)**	***P* Value**	**HR (95% CI)**	***P* Value**
**Age (years)** (< 50 vs. ≥ 50)	1.507 (0.855–2.656)	0.156			1.372 (0.808–2.328)	0.241		
**Gender** (Female vs. Male)	1.211 (0.577–2.541)	0.612			1.322 (0.679–2.571)	0.441		
**HBeAg positive** (No vs. Yes)	1.231 (0.849–1.484)	0.144			1.137 (0.614–2.103)	0.683		
**Liver cirrhosis** (No. vs. Yes)	1.339 (0.740–2.423)	0.335			1.442 (0.831–2.501)	0.193		
**AFP (ng/mL)** (< 400 vs. ≥ 400)	1.366 (0.805–2.318)	0.248			1.530 (0.939–2.495)	0.088		
**Tumor Size (cm)** (< 5 vs. ≥ 5)	2.535 (1.352–4.755)	**0.004**	1.916 (1.016–3.612)	**0.045**	2.078 (1.211–3.565)	**0.008**	1.618 (0.932–2.813)	0.088
**Tumor number** (Single vs. multiple)	1.556 (0.845–2.865)	0.156			1.541 (0.862–2.756)	0.145		
**Tumor satellites** (No vs. Yes)	1.387 (0.754–2.552)	0.292			1.392 (0.803–2.416)	0.237		
**Microvascular invasion** (No vs. Yes)	2.861 (1.554–5.265)	**0.001**	2.183 (1.182–4.032)	**0.013**	2.629 (1.531–4.514)	** < 0.001**	2.192 (1.260–3.812)	**0.005**
**Tumor differentiation** (Well vs. Intermediate)	1.967 (0.481–8.047)	0.347			1.566 (0.717–7.193)	0.103		
**Child–Pugh grade** (class A vs. B)	1.268 (0.626–2.566)	0.510			0.943 (0.470–1.890)	0.868		
**HBx expression** (low vs. high)	3.849 (2.291–6.468)	** < 0.001**	2.222 (1.307–3.777)	**0.003**	2.941 (1.856–4.659)	** < 0.001**	1.811 (1.110–2.953)	**0.017**
**CD68 expression** (low vs. high)	5.248 (3.160–8.713)	** < 0.001**	3.935 (2.343–6.606)	** < 0.001**	4.729 (2.667–7.540)	** < 0.001**	3.709 (2.251–6.112)	** < 0.001**

*AFP* α-fetoprotein, *HBx* hepatitis B virus X protein, *HR* hazard ratio, *CI* confidence interval

### Concomitant high HBx and CD68 expression was correlated with more unfavorable clinical features and worse outcomes of HBV-related HCC

**Fig. 3:**
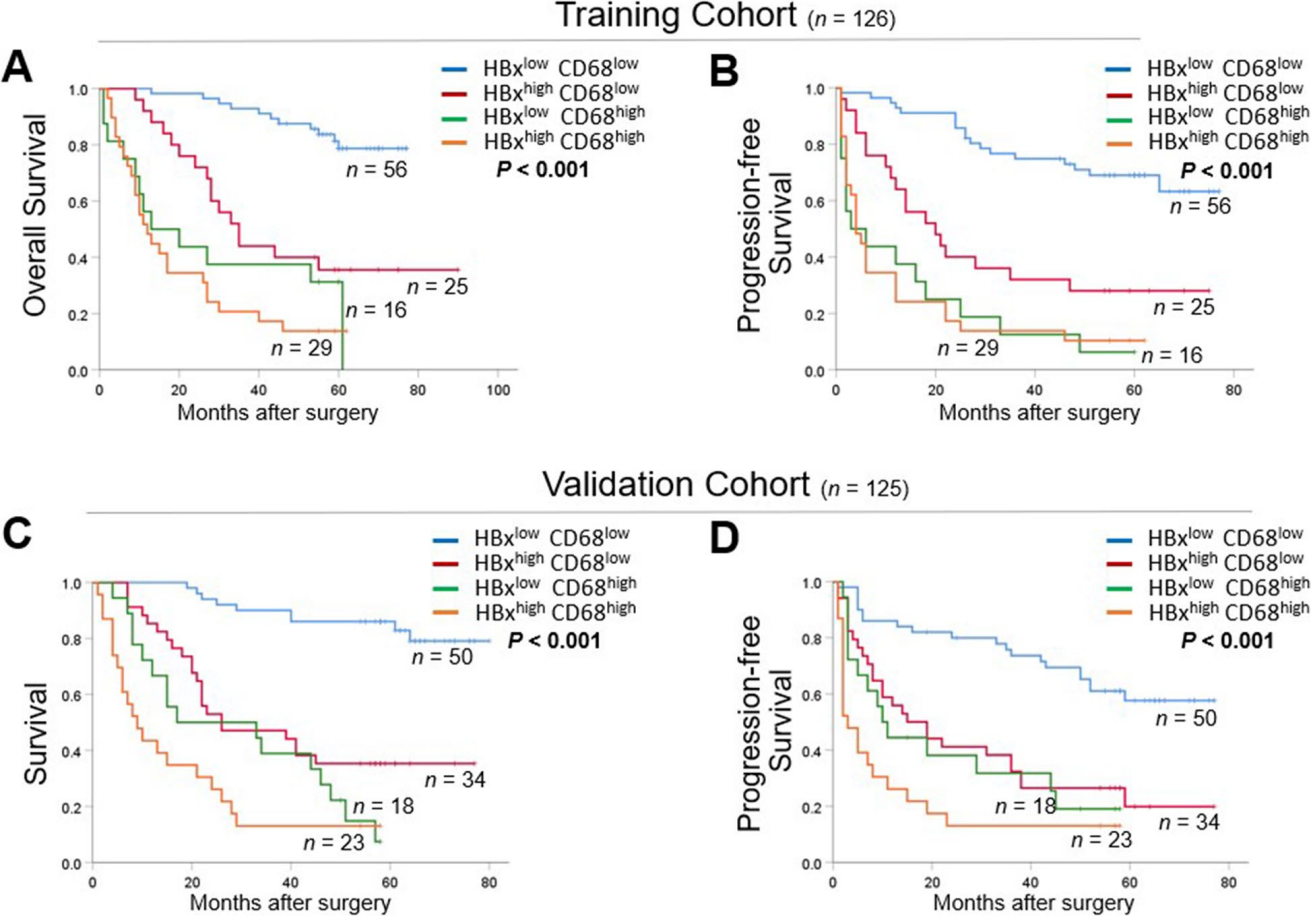
Integrating HBx and CD68 expression better indicated postoperative prognosis of patients with HBV-related HCC. **A**, **B** Kaplan–Meier curves of OS and PFS among 4 subgroups stratified by expression levels of HBx and CD68 (both low, both high and either high) in training cohort (*n* = 126) and **C**, **D** in validation cohort (*n* = 125). OS, overall survival; PFS, progression-free survival

To further investigate the potential synergistic effect of combining HBx and CD68 expression in prognostic prediction of HBV-related HCC, all patients from the training cohort were stratified into 4 groups based on the cut-off values of HBx and CD68 from the ROC curves: I: Both low (*n* = 56); II and III: either HBx or CD68 high (*n* = 25 and 16); IV: both high (*n* = 29). Table 2 summarizes the clinicopathological features among the 4 groups of patients in the training cohort. Comparison exhibited that patients with both high expression of HBx and CD68 were more often had MVI and a larger tumor size (both *P* < 0.01). The results comparing long-term survival outcomes among the different groups are depicted in Fig. 3A and B. As expected, the HBX^high^CD68^high^ group had worst OS and PFS than the remaining subgroups. Moreover, repeated analyses in the validation cohort also showed similar results as above, as the most aggressive clinical characteristics and shortest long-term survival time were seen in patients with concomitant high HBx and CD68 expression (Fig. 3C, D; Supplementary Table 3). Taken together, these data suggested that the combination of HBx expression and CD68 could effectively stratify the prognosis and thus had great potential to predict unfavorable clinical outcomes in HCC patients relating to HBV infection.

**Table 2:** Clinicopathological characteristics of HCC subtypes defined by HBx and CD68 expression in training cohort (*n* = 126)

**Characteristics**	**HBx / CD68 expression**					
	**Both low** **(*n* = 56)**	**HBx**^**high**^ **CD68**^**low**^ **(*n* = 25)**	**HBx**^**low**^ **CD68**^**high**^ **(*n* = 16)**	**Both high** **(*n* = 29)**	**Total** **(*n* = 126)**	*P* value*
**Age (years)**						0.856
< 50	27	15	9	14	65	
≥ 50	29	10	7	15	61	
**Gender**						0.035
Male	52	25	12	23	112	
Female	4	0	4	6	14	
**HBeAg positive**						0.105
Yes	6	6	5	13	30	
No	50	19	11	16	96	
**Liver cirrhosis**						0.721
Yes	40	19	13	22	94	
No	16	6	3	7	32	
**AFP (ng/mL)**						0.787
> 400	36	15	11	21	83	
≤ 400	20	10	5	8	43	
**Tumor size (cm)**						0.001
< 5	28	2	2	6	38	
≥ 5	28	23	14	23	88	
**Tumor number**						0.621
Single	47	22	14	22	105	
Multiple	9	3	2	7	21	
**Tumor satellites**						0.125
Yes	41	16	15	24	96	
No	15	9	1	5	30	
**Microvascular invasion**						< 0.001
Yes	26	20	14	22	82	
No	30	5	2	7	44	
**Tumor differentiation**						0.542
Well (I)	3	2	1	0	6	
Intermediate (II-III)	53	23	15	29	120	
**Child–Pugh grade**						0.259
Class A	52	21	12	25	110	
Class B	4	4	4	4	16	
**TNM stage**						0.020
I	27	5	3	5	40	
II	26	18	12	19	75	
III	3	2	1	5	11	

*HCC* hepatocellular carcinoma, *AFP* α-fetoprotein, *TNM* Tumor Node Metastasis

^*^Statistical significance was calculated by chi-square or fisher's exact test for categorical/binary measures

### Both high expression of HBx and CD68 in combination with clinical variables indicated a better prognostic stratification in patients with HBV-related HCC

**Fig. 4:**
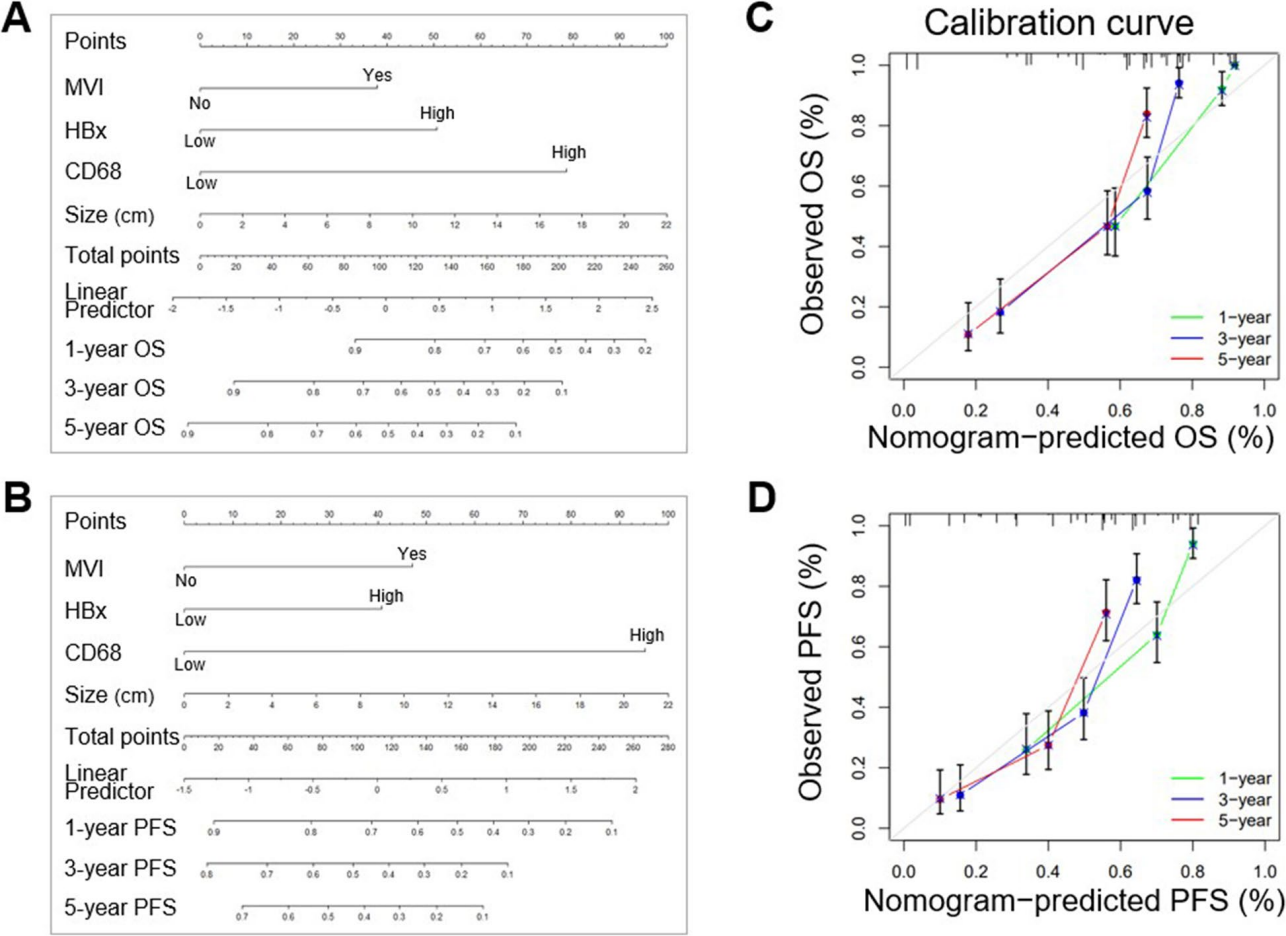
The established nomograms showed good predictive ability for OS and PFS in patients with HBV-related HCC. **A**, **B** The nomogram models for prediction of 1-, 3-, and 5-year OS and PFS for patients receiving liver resection for HBV-related HCC in training cohort. OS, overall survival; PFS, progression-free survival. **C**, **D** Calibration curves of the nomogram for predicting 1, 3, and 5-year OS and PFS

Next, the prognostic accuracy of either HBx, CD68, or clinical parameters alone or in combination was assessed by using C-index analysis. Table 3 shows the combination of HBx and CD68 expression to be markedly superior to any of the indicators alone, while the incorporation of HBx and CD68 into currently established clinical parameters presented the best prediction accuracy with the highest C-index value for post-surgical survival prediction in patients with HBV-related HCC. Similarly, in the validation set, the C-indexes of the constructed model were 0.869 (95% CI 0.805–0.933) and 0.811 (95% CI 0.730–0.893) for OS and PFS, respectively (Supplementary Table 4). On the basis of abovementioned results, an optimal nomogram model integrating HBx, CD68 and clinical variables (MVI and tumor size) was further constructed to better stratify the postoperative prognosis (Fig. 4A and B). Calibration curves for the models exhibited good performance in estimating the 1, 3, and 5-year OS and PFS (Fig. 4C and D), which meant the nomogram-predicted survival probabilities well matched the actual survival rates.

**Table 3:** C-index analyses of the prognostic accuracy of HBx, CD68 and clinical variables alone or in combination for overall survival and progression-free survival in training cohort (*n* = 126)

Characteristics	C-index (95% CI)	
	**Overall survival**	**Progression-free survival**
**Tumor size** (< 5 vs. ≥ 5)	0.616 (0.517 ~ 0.715)	0.604 (0.501 ~ 0.707)
**MVI** (No vs. Yes)	0.648 (0.552 ~ 0.745)	0.665 (0.565 ~ 0.765)
**HBx** (low vs. high)	0.715 (0.624 ~ 0.807)	0.684 (0.589 ~ 0.778)
**CD68** (low vs. high)	0.725 (0.634 ~ 0.815)	0.722 (0.637 ~ 0.814)
**Tumor size + MVI**	0.702 (0.610 ~ 0.794)	0.715 (0.622 ~ 0.808)
**HBx + CD68**	0.809 (0.732 ~ 0.887)	0.797 (0.716 ~ 0.877)
**HBx + Tumor Size + MVI**	0.783 (0.701 ~ 0.864)	0.763 (0.677 ~ 0.849)
**HBx + CD68 + Tumor Size + MVI**	0.835 (0.762 ~ 0.907)	0.826 (0.754 ~ 0.898)

*HBx* hepatitis B virus X protein, *MVI* microvascular invasion, *CI* confidence interval

### Comparisons of the HBx-CD68-based prognostic model with several conventional staging systems

The survival predictability was also compared between the proposed prognostic model with other commonly-used conventional models, including the TNM and Child–Pugh staging systems. The Kaplan–Meier curves for long-term outcomes according to the TNM and Child–Pugh stages for both training and validation cohorts are depicted in Supplementary Fig. 1. Comparisons of the predictive abilities for survival among these models are shown via the ROC curves, and the AUCs of this HBx-CD68-based model in the training set were superior to other well-established classifications for OS and PFS (all *P* < 0.001) (Supplementary Fig. 2). The above results were also verified in the validation set, suggesting that the proposed model performed better and was more accurate than other commonly-used staging systems in predicting long-term survival of HBV-related HCC following hepatectomy. Collectively, these results indicated that integrating tumor-specific biomarkers (HBx and CD68) with well-established clinical indicators could serve as a promising and reliable prognostic model with best predictive accuracy than the currently used single biomarker or clinical indicator for survival estimation.

## Discussion

In China, HBV-related HCC is the most common type of liver cancer and remains a highly lethal disease with limited response to current treatment modalities and dismal overall prognosis. Although enormous putative biomarkers have been identified for prognosis prediction of HCC patients [29, 30], most have very limited predictive power and only a few of them were independently validated in clinical settings. Thus, there remains an urgent demand to develop novel prognostic biomarkers or even integrated models to add further value in risk stratification and survival estimation for patients with HBV-related HCC.

HBV replication is closely linked to the occurrence and development of HCC in HBV epidemic areas [31], and HBx has been broadly indicated as a critical pathogenic protein for HBV-driven liver carcinogenesis [32]. Our group as well as others have been working toward the molecular mechanisms of HBx-mediated hepatocarcinogenesis for a long time and have elucidated the signaling pathways and downstream regulators involved in this process [10, 11, 33]. Meanwhile, elevated HBx expression was also predictive of adverse clinicopathological characteristics and poor outcomes, making it a promising prognostic indicator for HBV-related HCC evaluation. In addition, with increasing awareness of the great importance of immune microenvironment in HCC tumorigenesis, it therefore seems necessary to seek effective immunological biomarkers for identifying patients at high-risk of recurrence or even death following hepatectomy. However, very few studies have focused on the regulatory effect and mechanisms of HBx on the immune microenvironment in HCC, as well as a lack of investigation into the relationship between HBx expression and immune cell infiltration. Therefore, the present study for the first time investigated the clinical correlates of HBx expression with TAM infiltration and determined their prognostic values alone or in combination for HBV-related HCC, thus developing an integrated prognostic model including both intratumoral biomarkers and clinical variables, with an attempt to improve the prognosis assessment for surgical-treated HCC patients.

TAMs has gradually emerged as a research hotspot due to its great value in diagnosing and predicting tumor prognosis, with mounting evidence has linked the abundance of TAM subsets with patients’ clinical outcomes [17, 18]. In HCC, several studies have determined the association of CD68^+^ TAM infiltration and the long-term outcomes with conflicting results [34]. Specifically, high cell density of CD68^+^ macrophages in marginal area of the tumor was demonstrated to predict short OS and tumor recurrence after hepatectomy for HCC [35], whereas other groups supported that the intratumoral but not peritumoral CD68^+^ TAM infiltration correlated with tumor progression and unfavorable OS and PFS [21, 23, 36]. This was consistent with our findings, which suggested that infiltration of intratumoral CD68^+^ TAMs was an independent prognostic factor for HBV-related HCC patients. Therefore, it would be reasonable to use CD68 as specific TAM-related indicator to assess the patients’ outcomes after resection for HBV-related HCC in subsequent analyses.

Owing to the great heterogeneity of HCC, it appears difficult to comprehensively predict patients’ prognosis relying only on several specific intratumoral indicators. In addition to certain biomarkers, clinicopathological variables are easily accessible and have been widely applied to predict postoperative recurrence and clinical outcomes in HCC patients, including serum AFP level, tumor size, tumor number and the presence of MVI [4, 37]. In this study, by using multivariate Cox analysis, two commonly used clinical indicators (tumor size and MVI) were identified as independent predictors for survival and then were incorporated into the HBx-CD68-based prognostic model, thus providing more accuracy in the prediction of clinical outcomes for patients with HBV-related HCC. From our perspective, the main novelty of this study lies in revealing the potential correlation between oncogenic HBx and CD68^+^ TAMs infiltration, and also constructing an integrated prognostic model based on the possible link between HBx and TAM subset in HBV-related HCCs. As for the molecular mechanisms by which HBx regulates CD68^+^ TAMs in HCC, our previous findings revealed that HBx could promote stromal cell-derived factor-1 (SDF-1) secretion and exert downstream effects by binding to its ligand C-X-C chemokine receptor 4 (CXCR4) [11], while interaction with SDF-1 and CXCR4 was shown to drive recruitment and M2-type polarization of TAMs in HCC, thus inducing an immunosuppressive microenvironment and accelerating tumor progression [38]. These data may partially explain the close link between HBx expression and CD68^+^ TAM infiltration in HBV-related HCCs, and it is reasonable to speculate that HBx may regulate CD68^+^ TAMs infiltration in an SDF-1/CXCR4-dependent manner. It is hoped that our data may help establish a novel and reliable tool for survival prediction by simultaneously integrating both HBV-associated oncoprotein, TAM biomarker and routinely used clinicopathological parameters, which may partially compensate for the lack of effective prognostic biomarkers or models for HBV-related HCC at present.

In the present study, the prognostic value of our proposed model for HBV-related HCC was further compared with other HCC-specific biomarkers, like AFP, and conventional staging systems. Of note, although AFP was the most widely used serum biomarker for HCC diagnosis [39], its serum level as a continuous variable could not accurately predict post-surgical prognosis of HBV-related HCC patients, with AUCs of 0.550 and 0.565 for 5-year OS in the training and validation sets. In addition, nearly 20.3% of patients (51/251) in our overall cohort had normal AFP level, suggesting that any prognostic models including serum AFP value may not be accurate enough for survival estimation, especially for AFP-negative HCCs. Meanwhile, the Child–Pugh grading and TNM staging system are most commonly used for the evaluation of liver function and clinical outcomes in HCC patients [40, 41], but their predictive powers remain dismal due to the limited specific populations that are applied for [42]. For instance, reported prognostic accuracies of TNM stage for predicting long-term survival of HCC ranged from 0.590 to 0.656 depending on the examined cohorts [43, 44]. In the present study, our model exhibited favorable discriminatory capacities in both the training and validation sets and outperformed the conventional HCC staging systems in predicting postoperative survival. To some extent, this personalized model was established for patients who underwent liver resection for HBV-related HCC, and may give some hint for the estimation of individual prognosis following hepatectomy. At the same time, it must be acknowledged that, although the cell density of TAMs in HCC microenvironment was incorporated into this model, it still has limited potential to provide novel molecular therapeutic targets for HBV-related HCC or to assist clinicians in decision-making, especially can’t be used to identify those patients who may benefit from immunotherapies, which has been emerging as an important treatment modality for unresectable or recurrent HCCs in recent years. Therefore, whether our newly-established model could effectively predict the response to immunotherapy according to the risk stratification warrants further validation through more prospective clinical studies.

Certain limitations of this study should be acknowledged. First, since the data of this study was mainly collected from a Chinese cohort of patients with HBV-related HCC, this model may not be generalizable to HCC patients in Western countries, where chronic hepatitis C virus infection or alcoholic liver disease is the predominant etiology of HCC [2]. Second, because of the inevitable defects of retrospective design, much clinical information on whether the patients had received regular antiviral therapies or had developed HBV reactivation was missing, and these factors have been clearly stated to be involved in tumor recurrence of HBV-related HCC [4]. Third, due to the lack of a well-established external cohort, the external validation was performed by splitting overall cohort into training and validation sets at a ratio of 1:1, instead of using another independent cohort, which may partially influence the accuracy of our proposed predictive model. Fourth, although a strong correlation between HBx and CD68 expression was observed, the detailed regulatory mechanisms of HBx on CD68^+^ TAMs infiltration in HBV-related HCCs are lacking, and additional experiments are warranted to uncover the molecular basis of this correlation in the near future.

In conclusion, high HBx expression was significantly associated with more CD68^+^ TAMs infiltration in HBV-related HCC. Besides, combing this HBx-CD68-based classifier with clinicopathological indicators performed better than any indicator alone and showed superior predictability on long-term survival in patients with HBV-related HCC.

## Supplementary Information

**Additional file 1.**

## Data Availability

The datasets generated during and/or analyzed during the current study are available from the corresponding author on reasonable request.

## Abbreviations

HCC: Hepatocellular carcinoma
HBV: Hepatitis B virus
HBx: Hepatitis B virus X
TAM: Tumor-associated macrophage
HBsAg: Hepatitis B surface antigen
HbeAg: Hepatitis B envelope antigen
AFP: α-Fetoprotein
MVI: Microvascular invasion
OS: Overall survival
PFS: Progression-free survival
TNM: Tumor Node Metastasis
CT: Computed tomography
MRI: Magnetic resonance imaging
IHC: Immunohistochemistry
TMA: Tissue microarray
SDF-1: Stromal cell-derived factor-1
CXCR4: C-X-C chemokine receptor 4
ROC: Receiver operating characteristic
AUC: Area under the curve of ROC
C-index: Concordance index
SD: Standard deviation
HR: Hazard ratio
MV: Multivariable
CI: Confidence interval
