# Supplementary material for: Integrated analysis of intratumoral biomarker and tumor-associated macrophage to improve the prognosis prediction in cancer patients
Source: BMC Cancer. 2023 Jun 27;23:593. doi: 10.1186/s12885-023-11027-6 (PMC10294501; doi:10.1186/s12885-023-11027-6)
Supplement: Supplementary file 1 — Additional file 1. [file 12885_2023_11027_MOESM1_ESM.docx]

**Supplementary Table 1. Clinicopathological characteristics of patients with HBV-related HCC**

| **Characteristics** | **Training cohort**  **(*n* = 126)** | **Validation cohort**  **(*n* = 125)** | | **Total**  **(*n* = 251)** |
| --- | --- | --- | --- | --- |
| **Age (years)** |  | |  |  |
| < 50 | 65 | | 54 | 119 |
| ≥ 50 | 61 | | 71 | 132 |
| **Gender** |  | |  |  |
| Male | 112 | | 108 | 220 |
| Female | 14 | | 17 | 31 |
| **HBeAg positive** |  | |  |  |
| Yes | 30 | | 25 | 55 |
| No | 96 | | 100 | 196 |
| **Liver cirrhosis** |  | |  |  |
| Yes | 94 | | 91 | 185 |
| No | 32 | | 34 | 66 |
| **AFP (ng/mL)** |  | |  |  |
| > 400 | 83 | | 76 | 159 |
| ≤ 400 | 43 | | 49 | 92 |
| **Tumor size (cm)** |  | |  |  |
| < 5 | 38 | | 41 | 79 |
| ≥ 5 | 88 | | 84 | 172 |
| **Tumor number** |  | |  |  |
| Single | 105 | | 103 | 208 |
| Multiple | 21 | | 22 | 43 |
| **Tumor satellites** |  | |  |  |
| Yes | 96 | | 81 | 177 |
| No | 30 | | 44 | 74 |
| **Microvascular invasion** |  | |  |  |
| Yes | 82 | | 80 | 162 |
| No | 44 | | 45 | 89 |
| **Tumor differentiation** |  | |  |  |
| Well (I) | 6 | | 5 | 11 |
| Intermediate (II-III) | 120 | | 120 | 240 |
| **Child-Pugh grade** |  | |  |  |
| Class A | 110 | | 114 | 224 |
| Class B | 16 | | 11 | 27 |
| **TNM stage** |  | |  |  |
| I | 40 | | 44 | 84 |
| II | 75 | | 66 | 141 |
| III | 11 | | 15 | 26 |

HBV, hepatitis B virus; HCC, hepatocellular carcinoma; AFP, α-fetoprotein; TNM, Tumor Node Metastasis.

**Supplementary Table 2. Univariate and multivariate Cox regression analysis of HBx, CD68 expression and clinical variables with**

**overall survival and progression-free survival in validation cohort (*n* = 125)**

| **Characteristics** | **Overall survival** | | | |  | **Progression-free survival** | | | |
| --- | --- | --- | --- | --- | --- | --- | --- | --- | --- |
|  | **Univariate** | | **Multivariate** | |  | **Univariate** | | **Multivariate** | |
|  | **HR (95% CI)** | ***P* Value** | **HR (95% CI)** | ***P* Value** |  | **HR (95% CI)** | ***P* Value** | **HR (95% CI)** | ***P* Value** |
| **Age (years)**  (<50 vs. ≥50) | 0.624  (0.309-1.260) | 0.188 |  |  |  | 0.748  (0.874-1.983) | 0.339 |  |  |
| **Gender**  (Female vs. Male) | 1.408  (0.718-2.759) | 0.320 |  |  |  | 1.082  (0.557-2.100) | 0.816 |  |  |
| **HBeAg positive**  (No vs. Yes) | 1.055  (0.585-1.902) | 0.859 |  |  |  | 1.227  (0.725-2.075) | 0.446 |  |  |
| **Liver cirrhosis** | 1.141 | 0.638 |  |  |  | 1.222 | 0.438 |  |  |
| (No. vs. Yes) | (0.658-1.979) |  |  |  |  | (0.736-2.029) |  |  |  |
| **AFP (ng/mL)**  (<400 vs. ≥400) | 1.377 | 0.210 |  |  |  | 1.413 | 0.138 |  |  |
|  | (0.835-2.270) |  |  |  |  | (0.895-2.233) |  |  |  |
| **Tumor Size (cm)**  (<5 vs. ≥5) | 3.720  (1.944-7.119) | **0.001** | 2.344  (1.196-4.593) | **0.013** |  | 3.512  (1.989-6.202) | **0.001** | 2.440  (1.348-4.418) | **0.003** |
| **Tumor number**  (Single vs. multiple) | 1.650  (0.929-2.932) | 0.088 |  |  |  | 1.638  (0.967-2.774) | 0.066 |  |  |
| **Tumor satellites**  (No vs. Yes) | 1.170 | 0.545 |  |  |  | 1.382 | 0.186 |  |  |
|  | (0.703-1.950) |  |  |  |  | (0.856-2.232) |  |  |  |
| **Microvascular invasion**  (No vs. Yes) | 3.800 | **<0.001** | 2.003 | **0.041** |  | 3.347 | **<0.001** | 2.107 | **0.009** |
|  | (2.029-7.115) |  | (1.030-3.894) |  |  | (1.969-5.692) |  | (1.202-3.694) |  |
| **Tumor differentiation** | 1.453 | 0.603 |  |  |  | 0.699 | 0.486 |  |  |
| (Well vs. Intermediate) | (0.356-5.935) |  |  |  |  | (0.256-1.914) |  |  |  |
| **Child-Pugh grade** | 1.270 | 0.550 |  |  |  | 2.079 | 0.049 |  |  |
| (class A vs. B) | (0.580-2.779) |  |  |  |  | (1.067-4.054) |  |  |  |
| **HBx expression**  (low vs. high) | 4.006  (2.284-7.025) | **<0.001** | 2.399  (1.438-4.003) | **0.001** |  | 2.560  (1.634-4.010) | **<0.001** | 1.878  (1.186-2.973) | **0.007** |
| **CD68 expression**  (low vs. high) | 3.523  (2.124-5.843) | **<0.001** | 3.775  (2.248-6.338) | **<0.001** |  | 2.805  (1.778-4.424) | **<0.001** | 1.969  (1.222-3.172) | **0.005** |

AFP, α-fetoprotein; HBx, hepatitis B virus X protein; HR, hazard ratio; CI, confidence interval.

**Supplementary Table 3. Clinicopathological characteristics of HCC subtypes defined by HBx and CD68 expression in validation cohort (*n* = 125)**

| **Characteristics** | **HBx / CD68 expression** | | | | |  |
| --- | --- | --- | --- | --- | --- | --- |
|  | **Both low**  **(*n* = 50)** | **HBx^high^**  **CD68^low^**  **(*n* = 34)** | **HBx^low^**  **CD68^high^**  **(*n* = 18)** | **Both high**  **(*n* = 23)** | **Total**  **(*n* = 125)** | ***P* value***** |
| **Age (years)** |  |  |  |  |  | 0.487 |
| < 50 | 20 | 14 | 10 | 10 | 54 |  |
| ≥ 50 | 30 | 20 | 8 | 13 | 71 |  |
| **Gender** |  |  |  |  |  | 0.110 |
| Male | 44 | 29 | 18 | 17 | 108 |  |
| Female | 6 | 5 | 0 | 6 | 17 |  |
| **HBeAg positive** |  |  |  |  |  | 0.421 |
| Yes | 10 | 6 | 6 | 3 | 25 |  |
| No | 40 | 28 | 12 | 20 | 100 |  |
| **Liver cirrhosis** |  |  |  |  |  | 0.490 |
| Yes | 33 | 26 | 15 | 17 | 91 |  |
| No | 17 | 8 | 3 | 6 | 34 |  |
| **AFP (ng/mL)** |  |  |  |  |  | 0.500 |
| > 400 | 28 | 21 | 10 | 17 | 73 |  |
| ≤ 400 | 22 | 13 | 8 | 6 | 48 |  |
| **Tumor size (cm)** |  |  |  |  |  | 0.012 |
| < 5 | 23 | 12 | 4 | 2 | 41 |  |
| ≥ 5 | 27 | 22 | 14 | 21 | 84 |  |
| **Tumor number** |  |  |  |  |  | 0.601 |
| Single | 41 | 29 | 16 | 17 | 103 |  |
| Multiple | 9 | 5 | 2 | 6 | 22 |  |
| **Tumor satellites** |  |  |  |  |  | 0.562 |
| Yes | 33 | 20 | 14 | 14 | 81 |  |
| No | 17 | 14 | 4 | 9 | 44 |  |
| **Microvascular invasion** |  |  |  |  |  | <0.001 |
| Yes | 21 | 24 | 15 | 20 | 80 |  |
| No | 29 | 10 | 3 | 3 | 45 |  |
| **Tumor differentiation** |  |  |  |  |  | 0.639 |
| Well (I) | 3 | 1 | 1 | 0 | 5 |  |
| Intermediate (II-III) | 47 | 33 | 17 | 23 | 120 |  |
| **Child-Pugh grade** |  |  |  |  |  | 0.342 |
| Class A | 48 | 31 | 15 | 20 | 114 |  |
| Class B | 2 | 3 | 3 | 3 | 11 |  |
| **TNM stage** |  |  |  |  |  | 0.011 |
| I | 27 | 11 | 4 | 2 | 44 |  |
| II | 18 | 19 | 12 | 17 | 66 |  |
| III | 5 | 4 | 2 | 4 | 15 |  |

***** Statistical significance was calculated by chi-square or fisher's exact test for categorical/binary measures.

HCC, hepatocellular carcinoma; AFP, α-fetoprotein; TNM, Tumor Node Metastasis.

**Supplementary Table 4. C-index analyses of the prognostic accuracy of HBx, CD68 and**

**clinical variables alone or in combination for overall survival and progression-free survival in validation cohort (*n* = 125)**

| **Characteristics** | **C-index (95% CI)** | |
| --- | --- | --- |
|  | **Overall survival** | **Progression-free survival** |
| **Tumor size** (<5 vs. ≥5) | 0.677 (0.580~0.773) | 0.695 (0.595~0.795) |
| **MVI** (No vs. Yes) | 0.695 (0.600~0.790) | 0.688 (0.587~0.788) |
| **HBx** (low vs. high) | 0.684 (0.590~0.779) | 0.665 (0.567~0.763) |
| **CD68** (low vs. high) | 0.726 (0.636~0.815) | 0.635 (0.536~0.733) |
| **Tumor size + MVI** | 0.758 (0.672~0.844) | 0.768 (0.679~0.856) |
| **HBx + CD68** | 0.827 (0.752~0.902) | 0.724 (0.631~0.817) |
| **HBx + Tumor Size + MVI** | 0.797 (0.717~0.878) | 0.801 (0.718~0.885) |
| **HBx + CD68+Tumor Size + MVI** | 0.869 (0.805~0.933) | 0.811 (0.730~0.893) |

HBx, hepatitis B virus X protein; MVI, microvascular invasion; CI, confidence interval.

**Supplementary Figure 1. Kaplan-Meier survival analyses stratified patients in both training and validation cohorts based on Child-Pugh grading and TNM staging systems**

**
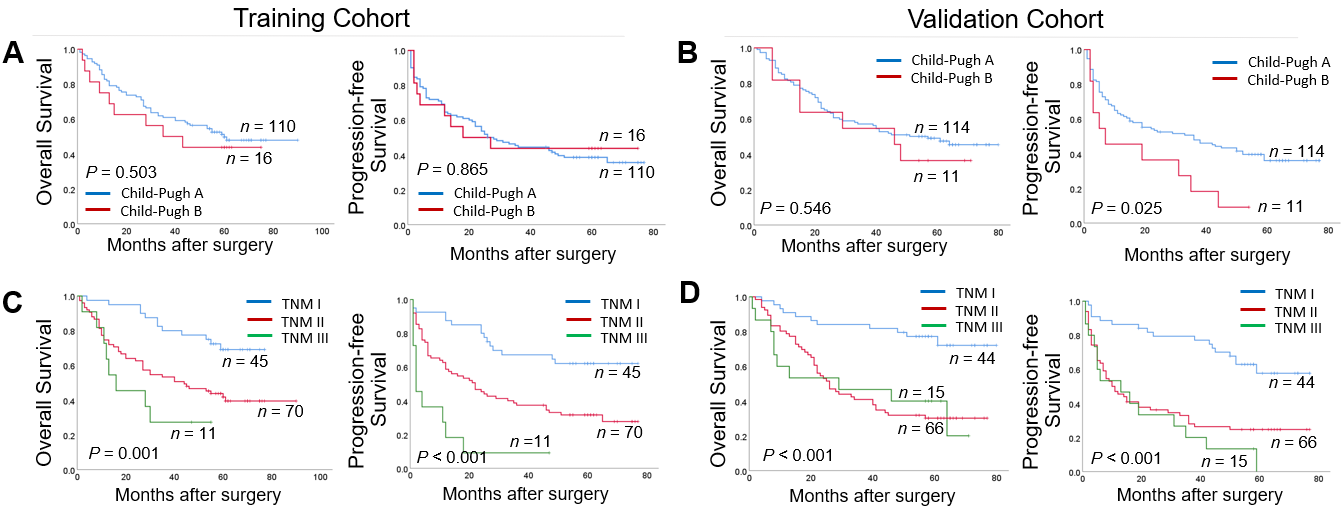
**

**(A-B)** Comparison of OS and PFS between HCC patients with Child-Pugh grade A versus grade B; **(C-D)** Comparison of OS and PFS among HCC patients with different TNM stages (I, II and III); *P* values were calculated by log-rank test. OS, overall survival; PFS, progression-free survival; TNM, Tumor Node Metastasis.

**Supplementary Figure 2. Receiver operating characteristic analyses for the accuracy of conventional staging systems versus the HBx-CD68-based prognostic model**

**in predicting postoperative survival for HBV-related HCC**

**
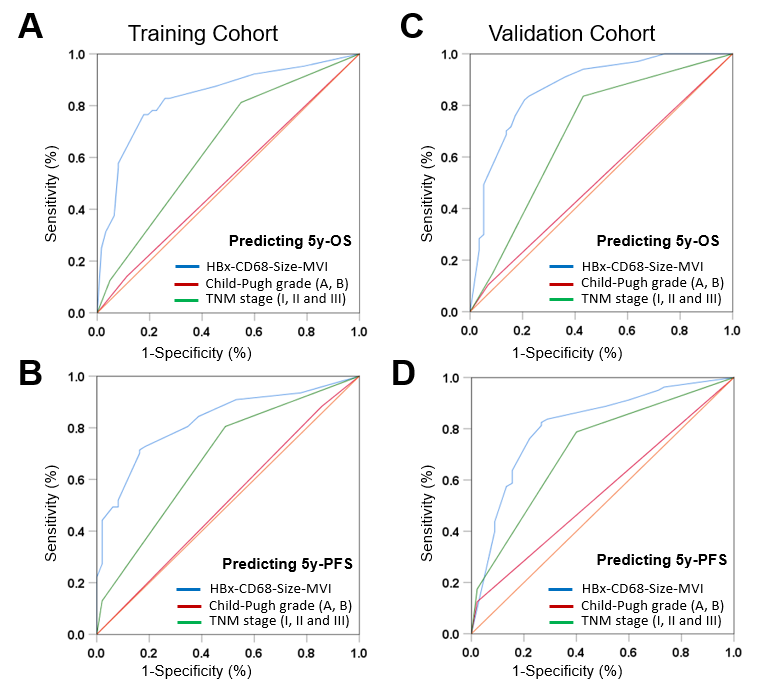
**

ROC curves for the performance of Child-Pugh grade, TNM stage and our prognostic model in predicting OS and PFS in training **(A-B)** and validation **(C-D)** cohort. AUC values for these indicators were presented and compared. HBV, hepatitis B virus; HCC, hepatocellular carcinoma; OS, overall survival; PFS, progression-free survival; TNM, Tumor Node Metastasis; ROC, receiver operating characteristic; AUC, area under the curve of ROC.
